# Dietary sodium intake and all-cause mortality in rheumatoid arthritis: an NHANES analysis (2003–2018)

**DOI:** 10.3389/fnut.2025.1518697

**Published:** 2025-05-30

**Authors:** Dongyi Li, Jiajun Li, Yiming Li, Ying Guan

**Affiliations:** ^1^Department of Orthopedic, The First Affiliated Hospital of Harbin Medical University, Heilongjiang, China; ^2^Department of Hepatic Surgery, The First Affiliated Hospital of Harbin Medical University, Heilongjiang, China; ^3^Department of Neurology, The First Affiliated Hospital of Harbin Medical University, Heilongjiang, China

**Keywords:** sodium intake, rheumatoid arthritis, all-cause mortality, NHANES, prospective cohort, nonlinear

## Abstract

**Background:**

In the field of nutritional epidemiology, the association between sodium intake and all-cause mortality in patients with rheumatoid arthritis (RA) remains inadequately explored. Consequently, the impact of sodium consumption on the prognosis of RA patients is not clearly defined, which leaves clinicians without adequate data to guide dietary sodium recommendations.

**Objectives:**

This study seeks to examine the potential relationship between sodium intake in the diets of patients with RA and all-cause mortality.

**Methods:**

A prospective cohort study analyzed 2,856 patients aged 20 and older with RA who participated in the National Health and Nutrition Examination Survey (NHANES) from 2003 to 2018. Comprehensive data on mortality, dietary sodium intake, and relevant confounding variables were systematically collected. Cox regression and restricted Cubic Splines (RCS) were employed to explore the potential associations.

**Results:**

After adjusting for confounding factors, a significant inverse correlation was observed between dietary sodium intake and the risk of all-cause mortality in patients with RA. When sodium intake was treated as a continuous variable, the hazard ratio (HR) was 0.68 (95% CI: 0.56–0.81, *p* < 0.001). When sodium intake was categorized into quartiles, compared to the lowest intake group Q1 (≤ 2.1 g/day), the HRs for Q2, Q3, and Q4 (2.1–2.8 g/day, 2.8–3.7 g/day, and ≥ 3.7 g/day) were 0.89 (95% CI: 0.75–1.06, *p* = 0.212), 0.74 (95% CI: 0.62–0.88, *p* = 0.001), and 0.70 (95% CI: 0.58–0.85, *p* < 0.001), respectively. The nonlinear model revealed a threshold effect, identifying a breakpoint at a sodium intake of 3.1 g/day. Below this threshold, for each additional unit of intake, the risk of all-cause mortality decreased by 14% (HR = 0.86).

**Conclusion:**

The findings of this study demonstrate a negative correlation between increased dietary sodium intake and all-cause mortality risk in patients with RA within a specific range. The threshold analysis identified a breakpoint at a sodium intake of 3.1 g per day, which is equivalent to approximately 7.9 grams of salt, exceeding the World Health Organization (WHO) recommendation of 5 grams of salt per day. These findings challenge the prevailing notion that higher sodium intake is always detrimental. This may offer valuable insights for developing dietary guidelines for RA patients.

## 1 Introduction

Statistics show that RA has become one of the most common chronic diseases in the world today, affects approximately 0.5–1.0% of the global population (1). It is characterized by systemic autoimmune disease and symmetrical synovitis, leading to joint pain, swelling, and varying degrees of joint dysfunction in patients. This chronic immune disorder significantly impacts patients’ work, study, and overall quality of life (2, 3). It has been reported that the mortality rate in patients with RA is approximately 54% higher than that of healthy individuals (4). In 2019, there were over 18 million patients with RA worldwide, including 1.07 million new cases. This substantial number undoubtedly adds to the burden on regions and countries, with projections indicating that the global incidence of new RA cases will increase by 1.4 times by 2040 (5).

On the other hand, it is estimated that the global average sodium intake is around 3,950 milligrams (172 millimoles) per person per day, which is equivalent to nearly 10 grams of salt (NaCl) per person. This sodium intake significantly exceeds the recommendations set by the WHO, which strongly advises that adults limit their sodium intake to less than 2 grams per day, corresponding to less than 5 grams of salt (6). Therefore, it is commonly understood that increased sodium intake as a dietary element is associated with a higher risk of disorders such as cardiovascular conditions. Similarly, some studies suggest that excessive sodium intake may be associated with the onset and progression of systemic lupus erythematosus (SLE) and RA, although there is a lack of high-quality research to support this (7). In contrast, some studies indicate that the intake of nutritional elements may modulate inflammation and immunity, potentially reducing disease activity in RA patients, slowing disease progression, and possibly decreasing the medication dosage required for treatment (8, 9). Furthermore, as a dietary element, insufficient intake of sodium may also increase the risk of adverse outcomes in RA (9).

So far, there is still controversy regarding whether excessive sodium intake promotes the development of RA. Furthermore, there is a lack of research on the impact of sodium as a dietary element on the prognosis of RA patients, and the relationship between sodium intake and mortality risk in RA patients remains unclear. Therefore, this study evaluated the association between sodium intake and mortality among adult patients with RA in the United States, aiming to provide a unique perspective for adjusting dietary habits and improving prognosis in RA patients.

## 2 Methods

### 2.1 Source of data and study cohort

The NHANES offers an extensive evaluation of the health and nutritional status of the U.S. population. Our prospective cohort study utilized data from 8 cycles of the NHANES database from 2003 to 2018, collecting information on sodium intake and rheumatic diseases, including arthritis and rheumatoid arthritis. The data we obtained were collected in accordance with the ethical standards set by the Institutional Review Board of the Centers for Disease Control and Prevention (CDC). The rights of all participants should be protected, and all subjects should be clearly informed about the procedures and objectives of the study before providing their informed consent.

**Figure 1:**
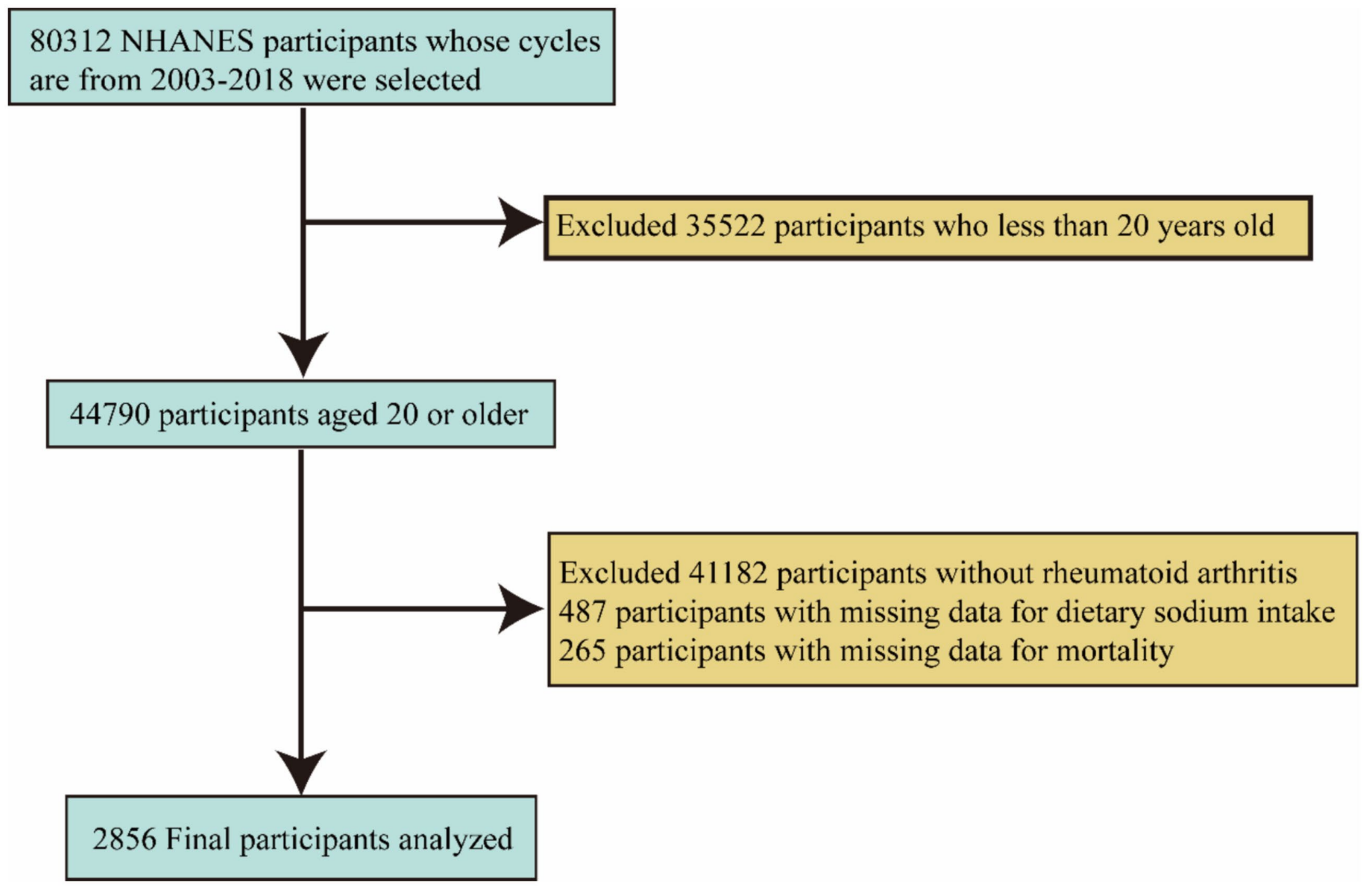
Flowchart of the patient selection process.

First, we excluded 35,522 individuals under the age of 20. Subsequently, we excluded an additional 41,182 individuals who were not diagnosed with RA, 487 individuals with missing sodium intake data, and 265 individuals with missing mortality data. Ultimately, the final sample size consisted of 2,856 participants. (Figure 1) illustrates the inclusion and exclusion criteria for the participants.

### 2.2 Definition of independent and dependent variables

NHANES supplied data from two 24-h dietary recall interviews for each participant. In our analysis, we determined the estimated sodium intake by averaging the data from the two dietary recall interviews. If one of the 24-h recalls was unavailable, we used the data from the single-day recall to estimate the intake.

The diagnosis of RA (10, 11) was determined using a two-stage self-administered questionnaire. First, participants were asked if they had ever received a diagnosis of arthritis from a healthcare professional, with responses limited to “yes” or “no.” Those who answered “yes” were then required to complete a second questionnaire, which asked for the specific type of arthritis diagnosed. This questionnaire also included additional options for “refuse” and “do not know” to account for any missing information. Ultimately, participants who identified their condition as “RA” were included in the RA population.

To ensure the accuracy and completeness of the data, all-cause mortality (12, 13) was used as the primary outcome measure, obtained from the NCHS Data Linkage - Mortality Data - Public-Use Files^1^. The follow-up time for each participant was defined as the period from the NHANES enrollment date to the date of death or until December 31, 2019, and was adjusted to accommodate balance analysis.

### 2.3 Covariates

In this study, based on clinical expertise and prior research, we selected several covariates related to RA for our analysis (14, 15). Age was categorized into two groups: ≤60 years and >60 years. Participants were classified by race/ethnicity as non-Hispanic white, non-Hispanic black, Mexican American, and other. Education level was divided into three categories: less than high school, high school or equivalent, and college or higher. Economic status was classified according to the poverty income ratio (PIR) into three categories: below 1.0 (low income), between 1.0 and 3.0 (middle income), and above 3.0 (high income). Marital status was categorized based on living arrangements with a partner, divided into two groups: married or cohabitating, and other (16). Body mass index (BMI), calculated as weight (kg) /height^2^ (m^2^), assesses weight status, with a higher BMI indicating greater obesity and categorizing individuals into three groups: < 25.0, ≥ 25 and < 30, and ≥ 30 (17). These reports were used to define whether participants had any of the aforementioned conditions. Smoking status was categorized into three groups: never smokers (defined as having smoked fewer than 100 cigarettes in their lifetime), current smokers (having smoked more than 100 cigarettes in their lifetime), and former smokers (having smoked more than 100 cigarettes in their lifetime and currently abstaining from smoking) (18). Physical activity status was categorized based on responses to the questionnaire regarding whether participants engaged in moderate work activity, classified as “yes” or “no.” Missing data were handled using multiple imputation, ensuring that no more than 20% of the data points remained unfilled.

### 2.4 Statistical analysis

In this study, all values were analyzed taking into account the complexity of the NHANES and incorporating sample weights to ensure accurate representation of the overall parameters of the population. Continuous variables were reported as weighted means accompanied by their corresponding 95% confidence intervals (CIs), whereas categorical variables were expressed as weighted percentages with their respective 95% CIs. We employed survey-weighted linear regression to assess intergroup differences for continuous variables and used survey-weighted chi-square tests to analyze differences in categorical variables. Sodium intake was categorized into quartiles, with the lowest 25% serving as the reference group to discuss transparency and its impact on risk assessment. Cox proportional hazards models were utilized, ensuring the validity of the model by checking the proportional hazards assumption. The model for covariate adjustment was described, providing adjusted hazard ratios (HR) and 95% CIs. Three multivariable models were constructed to explore this relationship, with each model sequentially adding different covariates.

A RCS model was established to examine the linear or nonlinear relationship between sodium intake and all-cause mortality in patients with RA. Threshold analyses were performed to provide detailed insight into the relationship between sodium intake and mortality and to determine threshold levels and verify the relationship between mortality and changes in sodium intake on either side of the threshold. Furthermore, Kaplan–Meier survival curves were plotted to visualize statistically significant differences in survival probabilities between different sodium intake quartiles. Subgroup analyses were performed to explore the variability in the effect of sodium intake on mortality, examining whether confounding factors affected the stability of the results. Statistical analyses were conducted using R software (4.3.1) and EmpowerStats, with statistical significance set at a two-sided *p* < 0.05.

## 3 Results

### 3.1 Study population characteristics

The study population consisted of 2,856 adults from the United States, with participants’ demographic and clinical characteristics stratified by sodium intake quartiles: Q1 (≤ 2.1 g/day), Q2 (2.1–2.8 g/day), Q3 (2.8–3.7 g/day), and Q4 (≥ 3.7 g/day). As shown in Table 1, there were statistically significant differences in sodium intake, age, sex, education level, family income poverty ratio, and BMI (*p* < 0.05). Participants with higher sodium intake were characterized by being male, younger than 60 years, having a higher BMI, higher education levels, and higher income levels. Additionally, sodium intake increased alongside rising BMI, education, and income levels within the population.

**Table 1:** Baseline characteristics of the study participants.

	Sodium intake level					*p*-value
	Total (*n* = 2,856)	Q1 (*n* = 714)	Q2 (*n* = 713)	Q3 (*n* = 715)	Q4 (*n* = 714)	
Sodium intake, g (CI)	3.13 (3.06, 3.20)	1.64 (1.60, 1.68)	2.45 (2.43, 2.47)	3.19 (3.17, 3.22)	4.81 (4.67, 4.94)	<0.001
Age group, % (CI)						<0.001
≤60	46.58 (44.14, 49.03)	33.99 (29.69, 38.58)	41.30 (36.20, 46.59)	48.46 (43.70, 53.25)	59.14 (54.16, 63.94)	
>60	53.42 (50.97, 55.86)	66.01 (61.42, 70.31)	58.70 (53.41, 63.80)	51.54 (46.75, 56.30)	40.86 (36.06, 45.84)	
Gender, % (CI)						<0.001
Male	36.21 (33.75, 38.73)	17.92 (14.26, 22.28)	23.05 (18.51, 28.32)	36.98 (32.24, 41.99)	61.04 (56.51, 65.39)	
Female	63.79 (61.27, 66.25)	82.08 (77.72, 85.74)	76.95 (71.68, 81.49)	63.02 (58.01, 67.76)	38.96 (34.61, 43.49)	
Race/ethnicity, % (CI)						0.709
Non-Hispanic white	76.28 (73.29, 79.02)	73.89 (68.94, 78.29)	75.89 (70.76, 80.37)	76.15 (72.82, 79.19)	78.54 (74.24, 82.29)	
Non-Hispanic black	10.46 (8.75, 12.46)	11.89 (9.21, 15.21)	10.89 (8.53, 13.81)	10.49 (8.38, 13.06)	8.97 (7.05, 11.34)	
Hispanic	4.71 (3.73, 5.93)	5.25 (3.57, 7.67)	3.92 (2.70, 5.66)	4.92 (3.51, 6.86)	4.81 (3.46, 6.65)	
Other	8.56 (7.21, 10.13)	8.98 (6.85, 11.67)	9.30 (6.03, 14.08)	8.43 (6.73, 10.52)	7.69 (5.63, 10.40)	
Education levels, % (CI)						0.005
Less than High school	20.60 (18.24, 23.18)	24.80 (20.30, 29.92)	21.53 (17.90, 25.67)	22.39 (18.74, 26.52)	15.04 (11.98, 18.71)	
High school or equivalent	26.18 (23.96, 28.54)	26.94 (22.37, 32.07)	28.17 (24.29, 32.40)	23.88 (19.91, 28.36)	25.86 (21.22, 31.12)	
College or above	53.21 (49.76, 56.64)	48.26 (42.32, 54.25)	50.30 (45.50, 55.10)	53.73 (48.82, 58.57)	59.10 (53.22, 64.73)	
PIR, % (CI)						0.015
<1.0	15.84 (13.75, 18.19)	21.03 (17.48, 25.08)	14.80 (11.50, 18.85)	15.69 (12.20, 19.94)	12.97 (10.10, 16.51)	
1.0–3.0	40.10 (37.33, 42.93)	40.41 (35.44, 45.58)	42.59 (37.87, 47.45)	38.90 (33.87, 44.18)	38.71 (34.20, 43.42)	
>3.0	44.06 (40.68, 47.49)	38.57 (33.37, 44.03)	42.61 (37.28, 48.12)	45.41 (39.56, 51.39)	48.32 (43.50, 53.18)	
Marital status, % (CI)						0.327
Married/living with partner	62.74 (59.75, 65.63)	58.33 (53.20, 63.29)	63.43 (57.89, 68.64)	64.16 (58.80, 69.18)	64.21 (58.90, 69.20)	
Other	37.26 (34.37, 40.25)	41.67 (36.71, 46.80)	36.57 (31.36, 42.11)	35.84 (30.82, 41.20)	35.79 (30.80, 41.10)	
BMI, %(CI)						0.002
< 25	24.10 (21.87, 26.48)	28.79 (24.18, 33.88)	25.45 (20.74, 30.83)	25.51 (21.73, 29.70)	18.11 (14.36, 22.59)	
25–30	31.82 (29.71, 34.00)	33.92 (29.54, 38.60)	32.27 (27.48, 37.45)	31.06 (27.25, 35.15)	30.48 (26.85, 34.37)	
≥ 30	44.08 (41.68, 46.52)	37.29 (33.10, 41.68)	42.28 (36.85, 47.90)	43.43 (39.31, 47.64)	51.41 (46.83, 55.95)	
Diabetes, % (CI)						0.881
Yes	22.90 (20.81, 25.14)	22.55 (18.93, 26.65)	23.04 (18.57, 28.22)	21.68 (17.70, 26.26)	24.11 (19.94, 28.84)	
No	77.10 (74.86, 79.19)	77.45 (73.35, 81.07)	76.96 (71.78, 81.43)	78.32 (73.74, 82.30)	75.89 (71.16, 80.06)	
Hypertension, % (CI)						0.079
Yes	60.26 (57.88, 62.59)	60.96 (56.85, 64.91)	58.62 (53.30, 63.74)	56.42 (51.27, 61.43)	64.53 (59.99, 68.83)	
No	39.74 (37.41, 42.12)	39.04 (35.09, 43.15)	41.38 (36.26, 46.70)	43.58 (38.57, 48.73)	35.47 (31.17, 40.01)	
Hyperlipemia, % (CI)						0.912
Yes	53.07 (50.50, 55.63)	54.55 (50.26, 58.77)	52.18 (46.89, 57.43)	52.43 (47.23, 57.57)	53.30 (48.53, 58.00)	
No	46.93 (44.37, 49.50)	45.45 (41.23, 49.74)	47.82 (42.57, 53.11)	47.57 (42.43, 52.77)	46.70 (42.00, 51.47)	
Smoking status, % (CI)						0.148
Current smokers	19.97 (16.88, 23.48)	22.54 (18.19, 27.58)	19.50 (15.52, 24.21)	16.79 (13.55, 20.63)	21.23 (16.90, 26.32)	
Former smokers	38.03 (33.95, 42.30)	39.92 (34.54, 45.55)	38.85 (33.37, 44.63)	38.90 (33.89, 44.15)	35.13 (29.05, 41.73)	
Never smokers	41.99 (38.21, 45.87)	37.54 (32.56, 42.79)	41.65 (36.00, 47.53)	44.31 (39.33, 49.41)	43.64 (37.82, 49.63)	
Moderate work activity, % (CI)						0.594
Yes	31.69 (29.16, 34.34)	32.50 (27.65, 37.75)	33.89 (28.49, 39.76)	31.41 (27.05, 36.12)	29.38 (24.94, 34.25)	
No	68.31 (65.66, 70.84)	67.50 (62.25, 72.35)	66.11 (60.24, 71.51)	68.59 (63.88, 72.95)	70.62 (65.75, 75.06)	

BMI, body mass index; PIR, poverty income ratio.

Q1: ≤ 2.1 g/day; Q2: 2.1–2.8 g/day; Q3: 2.8–3.7 g/day; Q4: ≥3.7 g/day.

### 3.2 Association between sodium intake and all-cause mortality in RA

Sodium intake was analyzed both as a continuous variable and as quartiles in three adjusted models to examine its relationship with all-cause mortality in RA patients, as shown in Table 2. In the fully adjusted Model 3, the regression analysis for the continuous variable indicated a HR of 0.68 (95% CI: 0.56–0.81, *p* < 0.001). Compared to the lowest sodium intake quartile (Q1, ≤2.1 g/day), the HRs for quartiles Q2, Q3, and Q4 (2.1–2.8 g/day, 2.8–3.7 g/day, and ≥3.7 g/day) were 0.89 (95% CI: 0.75–1.06, *p* = 0.212), 0.74 (95% CI: 0.62–0.88, *p* = 0.001), and 0.70 (95% CI: 0.58–0.85, *p* < 0.001), respectively. In both the continuous and categorical models, sodium intake indicated a significant negative correlation with all-cause mortality in RA patients.

**Table 2:** Association between sodium intake with all-cause mortality among participants with rheumatoid arthritis in the NHANES 2003–2018.

Sodium intake	Model 1			Model 2			Model 3		
	HR	95% CI	*p*-value	HR	95% CI	*p*-value	HR	95% CI	*p*-value
Continuous^a^	0.61	0.53, 0.71	<0.001	0.63	0.52, 0.75	<0.001	0.68	0.56, 0.81	<0.001
Categorical^b^									
Q1	Ref			Ref			Ref		
Q2	0.92	0.77, 1.09	0.325	0.87	0.73, 1.03	0.109	0.89	0.75, 1.06	0.212
Q3	0.73	0.63, 0.86	<0.001	0.70	0.59, 0.83	<0.001	0.74	0.62, 0.88	0.001
Q4	0.62	0.53, 0.74	<0.001	0.65	0.54, 0.79	<0.001	0.70	0.58, 0.85	<0.001
*p* for trend			<0.001			<0.001			<0.001

HR, hazard ratio; CI, confidence interval.

a Each 1 unit increase in sodium intake concentrations.

b Q1: ≤ 2.1 g/day; Q2: 2.1–2.8 g/day; Q3: 2.8–3.7 g/day; Q4: ≥3.7 g/day.

Model 1: Non-adjusted.

Model 2: Adjusted for sex, age, race, education, PIR, marital status.

Model 3: Adjusted for sex, age, race, education, PIR, marital status, BMI, diabetes, hypertension, hyperlipemia, smoking status, moderate work activity.

**Figure 2:**
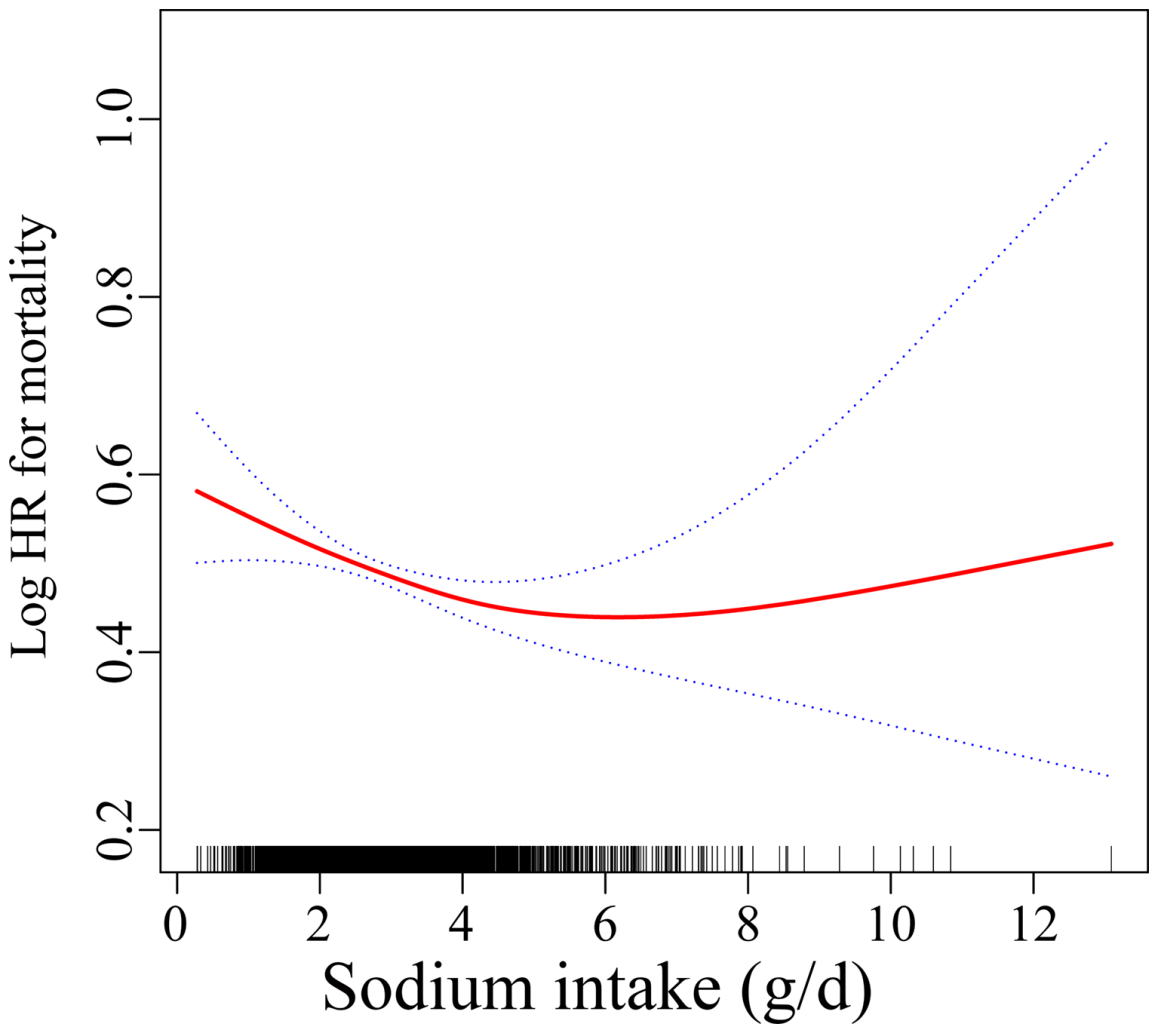
Association of sodium intake with all-cause mortality. Adjusted for sex, age, race, education, PIR, marital status, BMI, diabetes, hypertension, hyperlipemia, smoking status, moderate work activity.

In the RCS analysis, there is a nonlinear relationship between sodium intake and mortality rates (*p* < 0.001) (Figure 2). In the threshold analysis, we identified a turning point at 3.1 g/day. For participants with sodium intake below 3.1 g/day, the HR was 0.86 (95% CI: 0.80–0.92, *p* < 0.001) (Table 3), indicating that within this range, for every 1 g increase in daily sodium intake, the risk of mortality decreased by 14%. Conversely, in the population with sodium intake of 3.1 g or higher, there was no significant association between intake and mortality (*p* = 0.199). This result suggests that among individuals exceeding a daily intake of 3.1 g, the risk of mortality does not further decrease with increasing sodium intake.

**Table 3:** Threshold effect analysis of the relationship of sodium intake with all-cause mortality.

Sodium intake	Adjusted HR (95% CI)	*p*-value
Fitting by the standard linear model	0.89 (0.83, 0.95)	<0.001
Fitting by the two-piecewise linear model		
Inflection point	3.1 g	
Sodium intake < 3.1 g/dL	0.86 (0.80, 0.92)	<0.001
Sodium intake ≥ 3.1 g/dL	1.17 (0.92, 1.49)	0.199
*p* for Log-likelihood ratio		<0.001

Adjusted for age, sex, race, marital status, education, PIR, BMI, diabetes, hypertension, hyperlipemia, smoking status, moderate work activity. Only 99% of the data are displayed.

HR, hazard ratio; 95% CI, 95% confidence interval.

### 3.3 Subgroup analyses and interaction tests

**Figure 3:**
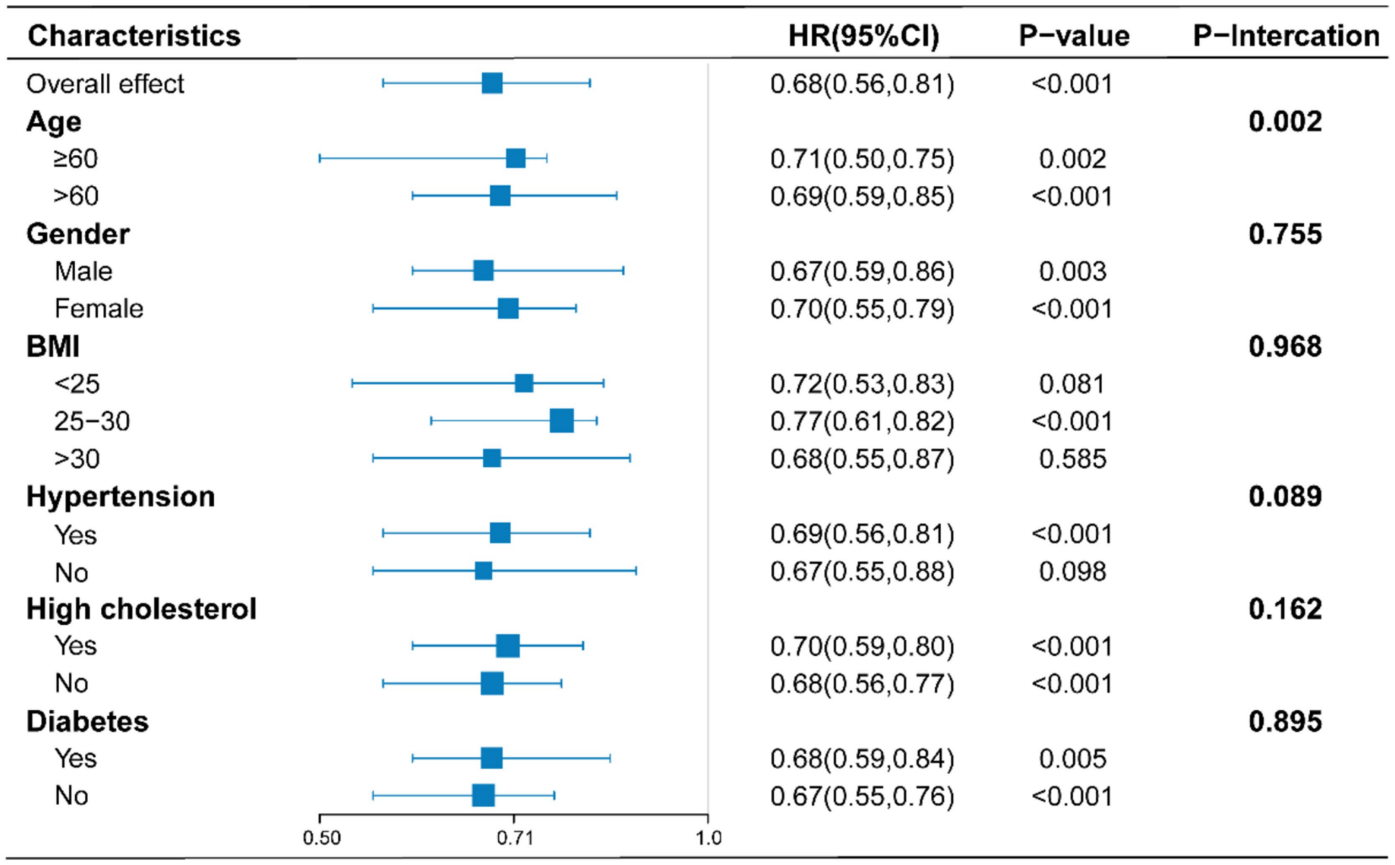
Stratified analyses of the association between sodium intake with all-cause mortality according to baseline characteristics. The *p* value for interaction represents the likelihood of interaction between the variable and sodium intake. Adjusts for: sex, age, race, education, PIR, marital status, BMI, diabetes, hypertension, hyperlipemia, smoking status, moderate work activity.

To assess potential factors influencing the relationship between sodium intake and all-cause mortality in RA patients, analyses were conducted within different subgroups stratified by age, sex, BMI, hypertension, hyperlipidemia, and diabetes. As shown in Figure 3, a negative correlation between sodium intake and all-cause mortality was observed in the majority of subgroups; however, this negative correlation was not statistically significant in populations with BMI < 25, BMI ≥ 30, and those without hypertension. In interaction tests, most covariates did not show significant interactions with each other (*p* > 0.05). Among the various subgroups, only age appeared to influence this relationship, while the overall negative correlation remained significant.

### 3.4 Kaplan–Meier curves

**Figure 4:**
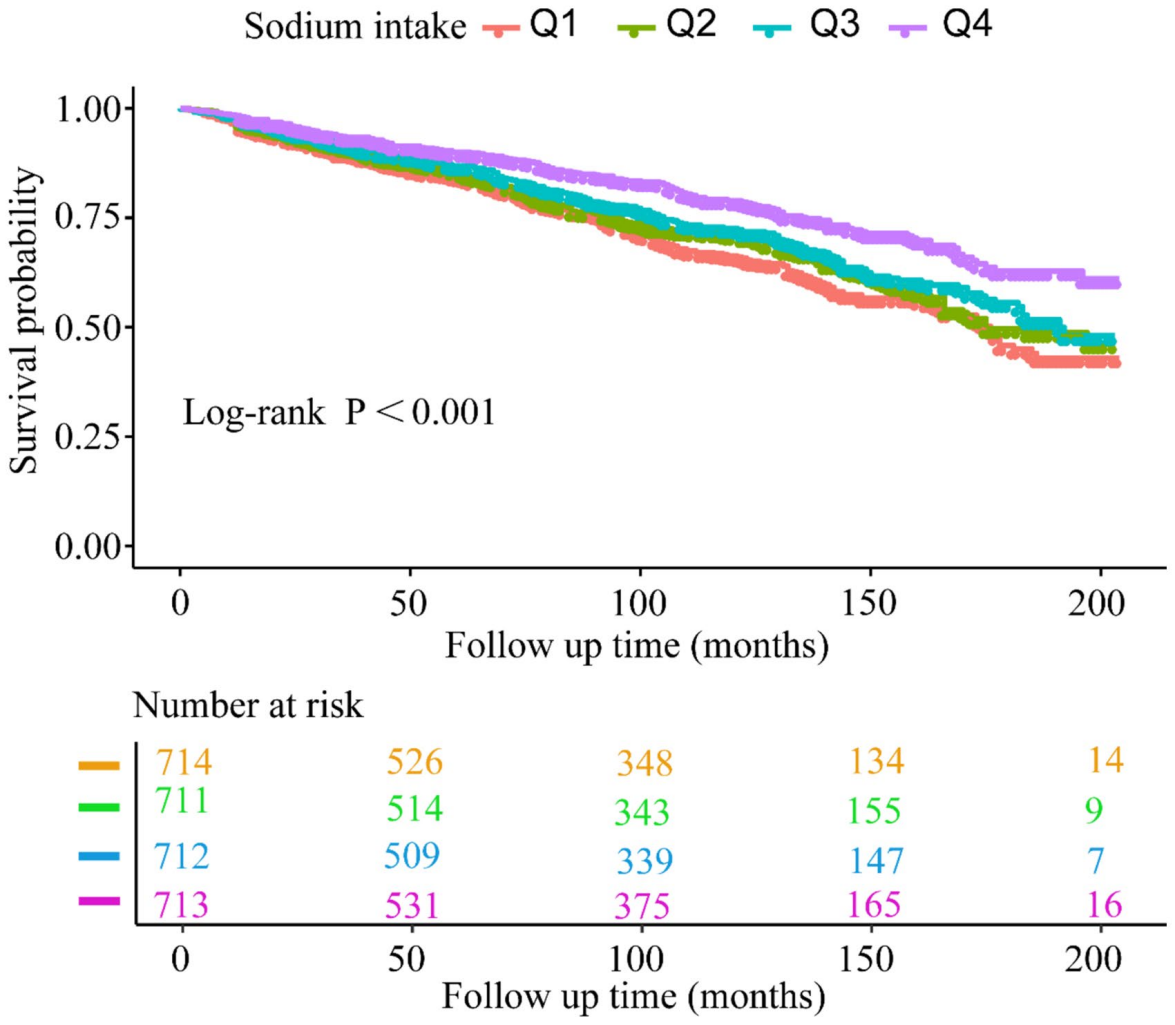
Kaplan–Meier survival rates for patients with RA in different sodium intake groups.

The Kaplan–Meier curves (Figure 4) indicate that the population with the highest sodium intake (Q4) had the highest all-cause survival rate. Additionally, we observed that survival rates decreased with lower baseline sodium intake (log-rank test: *p* < 0.001).

## 4 Discussion

Currently, there is a lack of research exploring the relationship between sodium intake and mortality in RA patients, leaving the association between dietary sodium consumption and long-term outcomes in this population unclear. In 2022, a study published in the prestigious cardiovascular journal European Heart Journal by Hao Ma et al. observed that adding salt to food more frequently was associated with an increased risk of early death and lower life expectancy, independent of dietary, lifestyle, and socioeconomic factors (19). Interestingly, in the same journal, Franz H. Messerli et al. conducted a statistical analysis using data from 181 countries. They found that increased sodium intake was associated with a longer average life expectancy and a reduction in mortality (20). Specifically, for every additional 1 g of sodium consumed per day (approximately 2.5 g of salt), life expectancy at birth increased by 2.6 years, and by 0.3 years at age 60, while all-cause mortality decreased by 131 per 100,000 individuals (20). This is roughly in the same direction as the result of the Kaplan–Meier curve in this study. Additionally, many recent studies (21, 22) have indicated that no significant association exists between sodium intake and all-cause mortality. Reducing sodium intake has not been shown to improve the risk of all-cause mortality or hospitalizations for cardiovascular reasons in heart failure patients (23). This undoubtedly challenges the long-held belief that sodium is a contributing factor to premature mortality. Conversely, lower sodium intake may be associated with a slight increase in the risk of cardiovascular diseases (24). Most of the world’s population consumes a moderate amount of dietary sodium (2.3–4.6 g/day), and intake within this range is not associated with an increased risk of cardiovascular diseases. It is only when sodium intake exceeds 5 g/day that the risk of developing cardiovascular disease begins to rise (25, 26). This assertion bears resemblance to the outcomes derived from the threshold analysis conducted in this study, which suggests that when sodium intake falls below 3.1 g, a rise in intake seems to confer a protective influence on all - cause mortality among RA patients.

The same controversies exist within the RA population. As an autoimmune disease, the immune mechanisms serve a vital function in the onset and progression of the disease (27). Multiple studies (28–31) suggest that high sodium intake can reverse the inhibitory effects on regulatory T cells (Tregs) and promote the differentiation of cells into pro-inflammatory helper T cell (Th)-1 and Th17 phenotypes. These effects are attributed to a cascade of events activated downstream of serum glucocorticoid kinase 1 (Sgk1), indicating that high sodium intake may be related to the onset of rheumatoid arthritis. Research by Nicola Wilck and colleagues supports the notion that sodium acts as an important regulator of immune responses through its effects on Th cell differentiation and function (32). They noted that sodium-induced immune modulation may have both beneficial and detrimental effects depending on the specific immune environment. For instance, sodium intake may help maintain normal immune system function by influencing the composition of the gut microbiota, thereby reducing the risk of autoimmune diseases (32). Rossana Scrivo and colleagues investigated the impact of dietary sodium intake on adaptive immunity, hypothesizing that dietary sodium could suppress Th17 cell levels while increasing Treg cell levels, ultimately leading to the inhibition of inflammatory responses in RA patients (33).

Although the relationship between sodium intake and the occurrence and progression of RA remains controversial, this study found a close association between dietary sodium intake and all-cause mortality in RA patients, demonstrating a statistically significant negative correlation within a certain range. In addition, emerging evidence from studies conducted within Chinese community - based populations has unveiled a U - shaped association between sodium intake and all - cause mortality. Specifically, both extremely low and excessively high sodium intake levels are linked to an elevated risk of all - cause mortality, whereas moderate sodium intake is associated with a reduced risk (34). It is notable that the results of several surveys conducted by O ‘Donnell M et al. that studied and utilized the British biological Database confirmed this view. These studies jointly indicate that the relationship between sodium intake and the risk of death is not a direct linear one; rather, it follows a nonlinear J-shaped or U-shaped pattern (35, 36). Furthermore, excessive restriction of sodium intake may increase the incidence and mortality of other diseases in the population (37, 38). Therefore, we suggest that maintaining sodium intake within a reasonable range may be a beneficial strategy for improving the outcomes of RA patients. This finding stands in direct opposition to the prevailing notion that high sodium intake, primarily attributable to salt consumption, is associated with a deterioration in outcomes for patients with RA. It necessitates a critical re - evaluation of the established dietary guidelines and therapeutic interventions targeting sodium modulation in this patient population. The threshold effect indicates an ideal sodium intake with a turning point around 3.1 g/day, approximately equivalent to 7.9 g of sodium chloride, which is below the global average intake of 10 g of sodium chloride (6). This implies that when formulating dietary plans, RA patients ought to take into account maintaining their total sodium chloride intake around the level of 7.9 grams. Such a targeted intake level may hold potential implications for optimizing health outcomes in this patient population.

Within the landscape of global research on RA, this study is the inaugural investigation into the relationship between sodium intake and all-cause mortality among RA patients. It offers crucial insights for clinicians in the dietary health management of this population. However, it also has some unavoidable limitations. Firstly, the diagnostic validity of RA in this database is inherently limited by its reliance on self-reported data derived from prior physician-provided diagnoses. This retrospective ascertainment approach introduces risks of case misclassification or underascertainment (e.g., recall bias from patient memory inaccuracies and selection bias from healthcare access disparities), thereby undermining the precision of outcome measures. Secondly, the absence of key common chronic disease variables and potential confounding factors may introduce bias into the findings. Future research should incorporate additional potentially relevant covariates to address these limitations. Lastly, the data in this study is exclusively derived from the American population, leaving the applicability of the findings to other racial groups uncertain. Ultimately, whether our conclusions apply to the broader human population requires larger-scale studies or randomized controlled trials for confirmation. Additionally, more in-depth research is required to explore the physiological mechanisms linking sodium intake to mortality risk in RA patients.

## 5 Conclusion

In conclusion, this study suggests that, within a specific range, increased sodium intake may be independently linked to all-cause mortality in individuals with RA, demonstrating a significant negative correlation. Increasing sodium intake appropriately may be an effective measure to improve the prognosis of RA. The threshold analysis identifies an optimal sodium intake level, with a breakpoint around 3.1 g/day, suggesting that sodium intake near this threshold reduce mortality in RA patients. This finding appears to run counter to the conventional wisdom, which posits that high sodium intake, primarily from salt, is associated with an elevated incidence of adverse outcomes in RA patients. This result may provide valuable insights for developing healthy dietary guidelines for RA patients. However, further investigation is required to substantiate the feasibility of our findings and to delve into the underlying mechanisms involved.

## Data Availability

The original contributions presented in the study are included in the article/supplementary material, further inquiries can be directed to the corresponding author.

## Footnotes

^1^ http://cdc.gov
